# Profile of diabetes and cardiovascular risk factors in adults Anjouan Island (Comoros)

**DOI:** 10.11604/pamj.2019.33.140.19016

**Published:** 2019-06-25

**Authors:** Rachmat Attoumane Ben Ali, Zineb Hannoun, Khouloud Harraqui, Lotfi Zeghari, Youssef Aboussaleh, Samir Mohamed, Mohamed Anssoufouddine, Abdellatif Bour

**Affiliations:** 1 Laboratoire des Essais Biologiques, Faculty of Sciences, Ibn Tofail University, Kenitra 14000, Morocco; 2 Laboratoire de Nutrition, Santé et Environnement, Faculty of Sciences, Ibn Tofail University, Kenitra 14000, Morocco; 3 Medical Service, Regional Hospital Center of Anjouan, Mutsamudu, Union of Comoros

**Keywords:** Prevalence, impaired fasting glucose, diabetes, risk factors, Anjouan, Comoros

## Abstract

**Introduction:**

The aim of this study was to estimate the prevalence of Diabetes Mellitus (DM) and Impaired Fasting Glucose (IFG) in the peri-urban adult population living in the island of Anjouan, Comoros and to investigate the factors associated with diabetes mellitus.

**Methods:**

The survey was a cross-sectional study, in which a sample of 902 individuals (540 women and 362 men) aged 25 to 64 was selected using empirical sampling “quotas” or “reasoned choice” survey method. Hypertension and obesity abdominal measurements of these subjects were collected during face-to-face interviews and following day fasting blood glucose was measured in capillary blood.

**Results:**

Participation rate was 83.5%. The mean age of subjects was 39.5 ± 11.63 years. The sex ratio was 0.67. Overall crude diabetes and IFG prevalence were 8.5% and 8.1%, respectively. The risk factors for diabetes type 2 onset were a family history of diabetes (P = 0.006), older age (P = 0.000), glycemic control (P = 0.010), excess waist circumference (P = 0.03) and hypertension (p = 0.000), were significantly positively associated with DM, contrary to sex (P = 0.142).

**Conclusion:**

These high figures confirm that diabetes and factors associated do not spare Anjouan population. Awareness, primary prevention, are to set up for a better control of non-communicable diseases.

## Introduction

Sub-Saharan Africa, like the rest of the world ongoing nutritional transition, is experiencing an increasing prevalence of diabetes alongside other non-communicable diseases [1]. The International Diabetes Federation suggest that diabetes affecting 285 million people around the world, with a projected rise to 438 million by 2030 [2]. Nowadays, diabetes has emerged as an important health problem in Africa [3, 4] and particularly in low- and middle-income countries [5], where steeper increase in the number of affected people [2]. Recent studies in urban populations suggest that the disease may now be more common in sub-Saharan Africa than previously thought [6]. In 2010, more than twelve million people were estimated to be living with diabetes in Africa and this is expected to increase to 23.9 million by 2030 [2]. The region faces to the double burden of communicable diseases (such as HIV and tuberculosis) and non-communicable diseases and their risk factors (such as diabetes and high blood pressure) [4]. The paucity constraints of data on diabetes from Africa limits considerably the development of potential preventative strategies. In Comoros, few prevalence investigations were conducted in the past. However, in 2011, the study carried by the Ministry of Health in partnership with the World Health Organization (WHO) showed that 4.8% and 5.2% of the population of, Comoros and Anjouan were diabetic respectively [7]. The aim of this survey is to determine an estimation of prevalence of diabetes mellitus, impaired fasting glucose and the risk factors associated to update previous data.

## Methods

**Sampling and description of population:** the investigation was a cross-sectional, descriptive and analytical study, carried out on the island of Anjouan, owned by the Comoros. Survey took place from July 16 to August 25, 2017. The target population is adults aged 25 to 64 living in Anjouan; excluding pregnant women, individual no resident in Anjouan and/or no consenting. According to forecasts of the General Census of Population and Housing carried out in the Comoros in 2003 [8]. A total of 107,365 people meeting our selection criteria resided on the island of Anjouan in 2017. A theoretical sample size of 864 was determined using the precision formula of the estimate of the confidence interval of the prevalence of diabetes mellitus. To do this, a margin of error of 2%, a confidence level of 95% and a maximum assumed prevalence value of 10% are considered, taking into account the evolution of the phenomenon based on prevalence figures and the risk factors from the 2011 STEPS Wise study. This theoretical size was then multiplied by the inverse of the participation rate, estimated at 80%, based on the experiences of surveys conducted in Anjouan in the field of health; which resulted in an estimated sample size of 1080 subjects included in this study. The selection of individuals in the sample was made using the empirical quota survey method with quota, sex and age criteria. Indeed, this quota method, chosen because of the unavailability of a frame, consisted of selecting a sample whose structure by sex and age is very close to that of the target population.

In the implementation of this method, we have selected, randomly with the help of the procedure of “Randomization” by using the ALEA function of the Ms Excel software, 20% of all the 91 localities of the island Anjouan. This gave 20 peri-urban localities of Anjouan randomly selected, so in a representative way. Then, the households to be surveyed were selected, in the field, according to the pre-established quotas according to the two criteria, sex and age. In the locality, the households were selected at random, following the direction of the tip of a pen, which guaranteed the representativeness of the selected households. Within the sampled household, the individual surveyed was selected using the KISH method [9], which gives the guarantee of the representativeness of the individuals surveyed. After obtaining informed consent, the subjects were submitted to a questionnaire. At the end of the interview, the subject was asked to fast after dinner to properly measure blood glucose the next day. One subject was declared absent after 2 visits or if he was traveling.

### Variables studied

**Diabetes occurrence:** early in the morning, after confirmation of the nocturnal fast of the subject, Capillary whole blood was obtained from a finger puncture and was immediately analysed using CONTOUR^®^Xt (BAYER) analyser. Diabetic patients were considered aware of their condition if they had been given the diagnosis by a health professional or if they reported the use of insulin or oral antidiabetic drugs. According to international standards [10], subject was considered diabetic if they had capillary whole blood glucose greater than or equal to 1.26g/l. impaired fasting glucose (IFG), where capillary whole blood glucose was between 1.10 and 1.25 g/l. Knowledge of diabetes, family history of diabetes and glycemic control were determined *via* questionnaire.

**Hypertension:** blood pressure (BP) was measured, using a TORM BRAS BP 3NZ1-3P monitor, in the sitting position, using the upper arm and an appropriately sized cuff after a 5-min rest period. Patients were considered to be hypertensive if they were being treated for known hypertension, or if their mean diastolic blood pressure was greater than or equal to 90 mmHg and their systolic blood pressure was greater than or equal to 140 mmHg [11].

**Abdominal obesity:** waist circumference were defined according to the latest IDF recommendations for Sub-saharan population (≥ 94cm for male and ≥ 80cm for female) [12].

**Statistical analysis:** data are expressed as proportions. The chi-square test, used for the confirmatory analysis of the independence between the distributions of the variables of the study. The level of statistical significance was set at P < 0.05. Database was obtained via a pretested questionnaire, then coded analysed on a data analysis software.

## Results

Out of a total of 1080 subjects selected, 902 subjects participated in the study, giving a response rate of 83.5%; ensuring the overall representativeness of the survey results. However, as in any study, the response rate differs. This was the case to measure blood glucose, whose response rate was 62.8% (567/902 persons). Women were the majority (540 against 362 men), respectively 59.9% against 40.1% (sex ratio 0.67), which is consistent with the overall structure of the Comorian population. The mean age of patients was 39.5 ± 11.63 years, with a range from 25 to 64 years. The age group 25-34 years (40.2%) was the most represented (Table 1).

**Table 1:** Description of the population studied

Gender	Number	Percentage (%)
Female	540	59.9
Male	362	40.1
**Age group (years)**		
25-34	363	40.2
35-49	329	36.5
50-64	210	23.3
**Total**	**902**	**100**

**Prevalence of diabete:** Table 2 shows the diabetes situation in the Anjouan population. The overall prevalence of diabetes mellitus (DM) was 8.5% (48 subjects) with 36 news detected cases and regarding IFG, prevalence was 8.1% (46 subjects). Altogether, disorder glycemic concerned 16.6% of population. The prevalence of diabetes mellitus was 5.3% for women against 3.2% in men (P = 0.11). As to age, was associated, significantly, to the increase in diabetes (P = 0.000) (Table 2). The average blood glucose level is 1.01 g/l (± 0.439) with a maximum of 5.26g/l.

**Table 2:** Prevalence of risk factors associated with diabetes (table only concerns individuals who have taken blood glucose)

Variables	Glycemic status							
	Normal		Impaired Fasting Glucose (IFG)		Diabete		Total Number	P
Gender	Number	Prevalence of normal subjects %	Number	Prevalence of IFG %	Number	Prevalence of Diabete %		
Female	290	51.1 %	35	6.2	30	5.3	355	0.142
Male	183	32.3%	11	1.9	18	3.2	212	
**Age group (years)**								
25- 34	193	34%	10	1.8	6	1.1	209	0.000
35- 49	171	30.2%	19	3.4	18	3.2	208	
50-64	109	19.2%	17	3	24	4.2	150	
**Knowledge of diabetes**								
Yes	358	63.1%	29	5.1	31	5.5	418	0.057
No	115	20.3%	17	3	17	3	149	
**Family history of diabetes**								
Yes	63	11.1%	4	0.7	11	1.9	78	0.006
No	293	51.7%	36	6.3	34	6	363	
Unclear	117	20.6	6	1.1	3	0.5	126	
**Glycemic control**								
Yes	62	10.9	6	1.1	14	2.5	82	0.010
No	411	72.5	40	7.1	34	6	485	
**Abdominal perimeter**								
Abdominal obesity	260	45.9	36	6.3	33	5.8	329	0.003
Normal	213	37.6	10	1.8	15	2.6	238	
HBP*								
Sane	282	49.7	17	3	17	3	316	0.000
Hypertensives	191	33.7	29	5.1	31	5.5	251	
**Total**	**473**	**83.4**	**46**	**8.1**	**48**	**8.5**	**567**	

* **HBP:** High blood pressure

### Cardiovascular risk factors

**Knowledge of diabetes** (locally known as “sugar disease”) was unknown in 29.2% (263/902 subjects) of total population. Table 2 shows the relationship between ignorance of the disease and diabetes. **Family history of diabetes** was found in 14.4% of total population (130 subjects). Of the 363 patients (subject who has measured blood glucose) which has responded “do not have a family history of diabetes,” 6.3% IFG; and 6% diabetes mellitus (statistically significant difference, P = 0.006), representing more 50% of all cases of diabetes mellitus found (Table 2). **Glycemic control:** 84.6% of total population (763 subjects) testified that they never measured their blood glucose. Of the 567 subjects who measured their blood glucose, the category that never tested blood glucose had the highest glycemic disorder, 6% and 7.1% of DS and IFG, respectively (statistically significant difference, P = 0.010) (Table 2).

The overall **prevalence of hypertension** in our study was high and scores 39.4% concerning 355 hypertensives. Hypertension was frequent in Women against in men (22.3% VS 17.1%) but, there were no statistically significant gender differences (P = 0.063). However, prevalence of hypertension showed a rising trend with increasing age (P = 0.000); particularly in those aged more 35 years. The average of the systolic and diastolic pressure was 130.28 (± 21.79) mm Hg and 72.08 (± 19.96) respectively (Table 3). **Abdominal obesity** had touched 54.5% of population representing 492 persons. It was more common in women than men (47.9% VS 6.7%; P = 0.000) (Table 3). Age was not associated with an increased prevalence of Abdominal obesity (P = 0.063). The mean waist circumference of the population is 89.34 ± 16.39 (female = 92.85 ± 17.36, male = 84.11 ± 13.20).

**Table 3:** Prevalence of High blood pressure and abdominal obesity of population by sex and age

Variable	Abdominal obesity				p	High blood pressure				p
	Yes		No			Yes		No		
**Gender**	**Number**	**%**	**Number**	**%**		**Number**	**%**	**Number**	**%**	0.063
Female	432	47.9	108	12	0.000	201	22.3	339	37.6	
Male	60	6.7	302	33.5		154	17.1	208	23.1	
**Age group**										
25-34	185	20.5	178	19.7	0.084	97	10.8	266	29.5	0.000
35-49	195	21.6	134	14.9		130	14.4	199	22.1	
50-64	112	12.4	98	10.9		128	14.2	82	9.1	

## Discussion

The subjects of this research were randomly selected from a representative sample of a predominantly female population (59.9% vs. 40.1%). The average age of our study is 39.5 years, similar to that reported by Djrolo *et al*. (39.4 years in Cotonou (Benin) in 2007) [13]. From our study emerges a prevalence of diabetes of 8.5% in Anjouan, attesting a worrying progression of the disease in Comorian population. In fact, in 2011, the STEPwise survey carried out by the Ministry of Health revealed a national rate of 4.8% (5.2% for Anjouan) [7] and in 2013, IFD reports a prevalence of 6.76% [14]. Prevalence of in IFG Anjouan was 8.1%. In addition, prevalence at the national level was 1.9% in 2011 [7]. This trend corresponds to the global evolution of the progression of the disease [15-17], particularly in low and middle countries income where three out of four people with diabetes live [18]. This is mainly due to the aging population, rapid and unplanned urbanization, the adoption of a sedentary lifestyle, particularly rich in saturated fatty acids diet, salt, sugar and rapid reduction of fiber intake [9, 19]. Several authors report the same upward trend, we cite the case of Djrolo *et al*. (From 3.3% in 2002 to 4.6% in 2007) in Benin [13] and Silva-Matos *et al*. (0.9% in 2005 to 3.3% in 2010) in Mozambique [20].

Moreover, our results corroborate with the literature, which evokes prevalences ranging from 2 to 20% in Africa [2, 21] and join those of Millogo *et al*. (8.5%) in Ouagadougou in 2015 [22]. In agreement with the literature, this prevalence is much higher than that reported in Morocco [23], Benin [24] and New Zealand [25]. In contrast, the prevalence found is much lower than those observed in other islands of the Indian Ocean, where the highest rates were recorded, with 16.4%, 16.2%, 12.2%, Reunion Island, Mauritius Island, Seychelles Island [26] and 10.5% in Mayotte [27], respectively. For gender, we found no statistically significant difference (P= 0.142), suggesting that the sex was not associated with an increased prevalence of diabetes; unlike some authors who observe a predominance in women [28] and others a reverse trend [29, 30]. Moreover all authors converge in the affirmation of the major influence of age on diabetes [24, 31, 32]. Elevated blood pressure, is a major factor in the development of diabetes and cardiovascular complications [33]. According to Foucarde *et al*. projection of hypertension rates in Africa-Saharan, in 2025, will varie between 15% and 33% [34]. With a prevalence of 39.4%, the Anjouan population far exceeds this interval placing them among the hypertensive populations such as Italy (38%), Algeria (36.5%) [35], Libya (42.5%) [35] and Senegal (46%) [36]. Beyond age factor (> 40 years) which is significant in the increase of arterial pressure [37], sex seems to be a trigger particularly in women (some authors have found the opposite [38-40] in excess of 40 years [41] where pregnancy, menopause, contraceptive pill use, and weight are involved [35, 38].

According literature, being overweight is a major factor in metabolic disorders. This work was mainly based on measuring the abdominal perimeter according to IFD criteria. The measure of waist circumference is used to identify android obesity, which high is usually accompanied by high blood pressure, dyslipidemia [42, 43] and is strongly correlated with the development of type 2 diabetes [43, 44]. Based on our results, android obesity is estimated to 54.5% of which 47.9% is recorded in women and 6.7% in men. This particular elevated levels in women is justified by the fact that the majority of this population still considers weight as a criterion of sumptuousness and comfort. In addition, Table 2 reaffirms the previous hypothesis. Our results clearly demonstrate that high waist circumference and high blood pressure are often accompanied by glycemic disturbance (Table 2). Among the high-risk behavioral factors mentioned in the literature is the abuse of tobacco. Indeed, chemical compounds of tobacco cause arterial stenosis, which leads to hypertension [36]. In our study, we report a total prevalence of 14.7% (result not reported in the article) and we find that one smoker out of two has high blood pressure and one diabetic out of two cumulates simultaneously the hypertension and the abdominal obesity. Besides to biological (age, sex, weight, family history) and behavioral (lifestyle and lifestyle) factors that significantly influence the development of diabetes, both elements play an essential role in the discovery and treatment of the latter, we quote ignorance of the disease and glycemic control which constitute, in the event of ignorance and/or negligence, major handicaps in the screening of the latter and therefore in treatment. Our study shows that many people are unaware of this condition, yet many are affected. Although the results of this study are descriptive, they highlight a worrying and alarming public health problem that should be considered quickly.

## Conclusion

In light of these results, it is obvious that diabetes and hypertension do not spare Anjouan population. The establishment of an information, treatment and monitoring system is therefore necessary. The urban and rural population is growing in semi-urban areas. This demographic transition is explained by the current economic difficulties in the country. These lead to the adoption of a lifestyle contrary to more protective ancestral habits and thus to the onset of diseases, locally known as Western diseases. Added to this is the stigmatization of diseases; In fact many individuals intentionally refuse to consult by embarrassment or refusal of the disease hence the urgent need for awareness within these environments.

### What is known about this topic

- Diabetes affects people worldwide and poses major public health and socioeconomic challenges;
- The projected growth for sub-Saharan Africa is 98%, from 12.1 million in 2010 to 23.9 million in 2030;
- Unless addressed, the mortality and disease burden from diabetes and other non-communicable disease (NCDs) will continue to increase.

### What this study adds

- This study has determined the exact prevalence of diabetes mellitus in the population of the island of Anjouan;
- Women are predisposed to glycemic disturbance as indicated in the literature;
- Our results inform us of the urgency of a non-communicable disease strategy if we want to stop their progression.

## Competing interests

The authors declare no competing interests.
